# Machine-learning-based tumor segmentation and classification using dynamic optical contrast imaging for thyroid cancer

**DOI:** 10.1117/1.BIOS.3.1.015001

**Published:** 2026-01-02

**Authors:** Tyler Vasse, Yazeed Alhiyari, Lauran K. Evans, Ramesh Shori, Maie St. John, Tuan Vo-Dinh

**Affiliations:** a Duke University, Biomedical Engineering Department, Durham, North Carolina, United States; b University of California, Los Angeles, David Geffen School of Medicine, Los Angeles, California, United States

**Keywords:** machine learning, cancer detection, optical imaging, intraoperative imaging, thyroid cancer

## Abstract

**Significance:**

Thyroid cancer is the most common endocrine malignancy, and diagnosis is often challenging due to overlapping features between benign and malignant nodules. Fine-needle aspiration, the clinical gold standard, frequently yields indeterminate results and lacks spatial context, leading to unnecessary surgeries. Real-time margin assessment also remains limited. There is a need for accurate, label-free, spatially resolved imaging. Dynamic optical contrast imaging (DOCI), which measures autofluorescence lifetimes of endogenous fluorophores, offers a promising platform for intraoperative cancer detection.

**Aim:**

We develop and evaluate a machine learning integrated DOCI framework to classify thyroid tissue subtypes and segment cancerous regions from *ex vivo* hyperspectral sections, with potential for real-time surgical use.

**Approach:**

Fresh *ex vivo* thyroid specimens were imaged using a 23-channel DOCI acquisition. A pixel-level principal component analysis (PCA) and logistic regression classifier produced tissue probabilities, aggregated by a regional majority-vote gate to categorize specimens as normal, follicular, or papillary. Tumor-specific squeeze-and-excitation U-Net models were trained on voxel-only inputs for semantic segmentation. PCA-guided channel ablation identified a reduced spectral subset, and the pipeline was retrained using a compact 12-channel input.

**Results:**

The first two PCA components explained over 70% of spectral variance and yielded well-separated tissue clusters. The regional PCA classifier achieved 92.3% validation accuracy and 100% accuracy on the test set. Full 23-channel U-Net models delivered strong segmentation (papillary: Dice nonempty 0.829, balanced Dice 0.914; follicular: Dice nonempty 0.618, balanced Dice 0.809). Reduced-channel models preserved most performance and improved follicular segmentation (Dice nonempty 0.762), confirming spectral redundancy.

**Conclusions:**

Integrating DOCI with interpretable machine learning enables accurate, label-free differentiation and segmentation of thyroid tissues. Channel reduction demonstrates that high performance is achievable with a compact spectral subset, supporting faster, more cost-efficient DOCI systems and future real-time intraoperative deployment.

Statement of Discovery This work uses machine learning combined with dynamic optical contrast imaging (DOCI), a label-free multispectral optical technique, to distinguish thyroid cancer subtypes based on endogenous tissue autofluorescence. This approach enables real-time, non-invasive tumor classification and margin assessment, with potential for intraoperative surgical guidance.

## 1 Introduction

Thyroid cancer is a significant health concern, representing the most common endocrine cancer worldwide, and continues to increase each year.^1^^,^^2^ This malignancy arises from the thyroid gland, a butterfly-shaped organ in the anterior neck that plays a crucial role in regulating metabolism through thyroid hormone production.^3^ Thyroid cancer encompasses several subtypes, including papillary, follicular, medullary, and anaplastic, each with distinct characteristics, treatments, and prognoses.^3^^,^^4^ Over recent decades, the incidence of thyroid cancer has been steadily increasing, largely attributed to advancements in diagnostic techniques and increased surveillance.^5^^,^^6^ This rise is particularly evident in papillary thyroid cancer, which has seen the most substantial growth in cases.^6^ Although the exact causes of thyroid cancer remain unclear, risk factors include exposure to ionizing radiation, family history, and certain genetic conditions.^3^^,^^7^

The complex anatomy of the neck region and the proximity of the thyroid gland to vital structures make accurate diagnosis and treatment challenging.^8^ This complexity further perplexes the differentiation between thyroid cancer and benign thyroid nodules, which affect up to 50% of individuals over the age of 50.^8^

Traditional diagnostic approaches for thyroid cancer often involve a combination of physical examination, blood tests, imaging studies, and fine-needle aspiration (FNA) biopsy. The FNA is considered the “gold standard” method for diagnosing thyroid nodules as benign versus malignant.^9^ However, these methods can sometimes lead to inconclusive results or unnecessary surgeries for benign nodules.^6^ In fact, 70% to 80% of thyroid nodules with indeterminate FNA results are ultimately found to be benign upon histological analysis of surgical specimens.^10^ In addition, FNA cannot provide spatial information about the specimens. Such spatial information is crucial during surgical removal, as positive tumor margins have significant prognostic implications.^11^ Currently, tumor margins are assessed through hematoxylin and eosin (H&E) staining after surgical resection, which are not performed during surgery to provide real-time feedback.

As such, there is a pressing need for advanced imaging techniques that can provide more accurate, spatially resolved, real-time, and detailed information about thyroid nodules, potential malignancy, and cancer subtypes.^8^ The development of novel imaging modalities has the potential to revolutionize the management of thyroid cancer by enabling earlier detection, more precise staging, better-informed treatment decisions, and real-time tumor margin assessment.^12^

Our team has previously developed and described DOCI, an innovative imaging approach that characterizes tissue by measuring its combined autofluorescence decay behavior. Human tissues contain numerous endogenous fluorophores, and their distinct decay signatures allow DOCI to noninvasively assess variations in tissue composition.^13^^–^^17^ In fresh *ex vivo* studies, we demonstrated that the DOCI system can effectively distinguish HNSCC from adjacent normal tissue and accurately localize tumors.^13^ This imaging method operates in real time, does not require the use of contrast agents, and provides a broad field of view during surgical procedures. By leveraging the fluorescence lifetime of various endogenous fluorophores within the tissue, DOCI generates a distinct molecular map to aid in identifying cancer margins. These margins are then validated against histological slides, which serve as the reference standard. Earlier studies have explored methods to statistically analyze DOCI images for tissue classification, employing approaches that range from basic logistic regression to advanced artificial neural networks.^18^

This work presents a unified analytical framework for detecting cancerous regions in DOCI-derived histopathologic images by integrating high-dimensional spectral acquisition with modern, interpretable machine learning methods. Our pipeline processes 23-channel DOCI autofluorescence data using principal component analysis (PCA) to reduce spectral redundancy and stabilize pixel-level representations. A pixel-wise classifier, based on PCA features and logistic regression with regional majority voting, provides robust specimen-level categorization into normal, follicular, or papillary classes. Tumor-specific squeeze-and-excite U-Net (SE-UNet) models then perform high-resolution semantic segmentation directly on voxel tensors, supported by extensive augmentation strategies to improve generalization across heterogeneous tissue samples.^13^^,^^19^ Channel-importance analysis further identifies the DOCI wavelengths most critical for accurate tumor detection, enabling compact reduced-channel models without major performance loss. By coupling diagnostic classification with precise spatial segmentation, this approach reliably differentiates benign from malignant tissue and captures subtype-specific tumor morphology, offering a pathway toward real-time, label-free decision support in thyroid cancer diagnosis and surgical management.^20^

## 2 Methods

### 2.1 Study Design, Specimens, and Imaging Acquisition

A retrospective study was conducted using fresh, *ex vivo* thyroid tissue collected intraoperatively under standard surgical and pathology workflows (n=72 cases). Immediately after excision, each specimen was cryosectioned (5  μm, unstained) and imaged using the DOCI platform prior to conventional hematoxylin–eosin (H&E) staining of adjacent sections. The H&E slides served as histopathological ground truth for diagnostic labeling.

An expert head-and-neck pathologist assigned case-level diagnoses as healthy (normal), follicular, papillary, or anaplastic carcinoma. Pixel-level tumor annotations were manually drawn on whole-slide images and exported as XML polygons, which were rasterized into binary ground-truth segmentation masks. Anaplastic cases, owing to their rarity and high intratumoral heterogeneity, were excluded from PCA-based pixel classification to prevent confounding between high-grade and differentiated thyroid phenotypes, though they were included during segmentation training for cross-class generalization analysis.

**Fig. 1:**
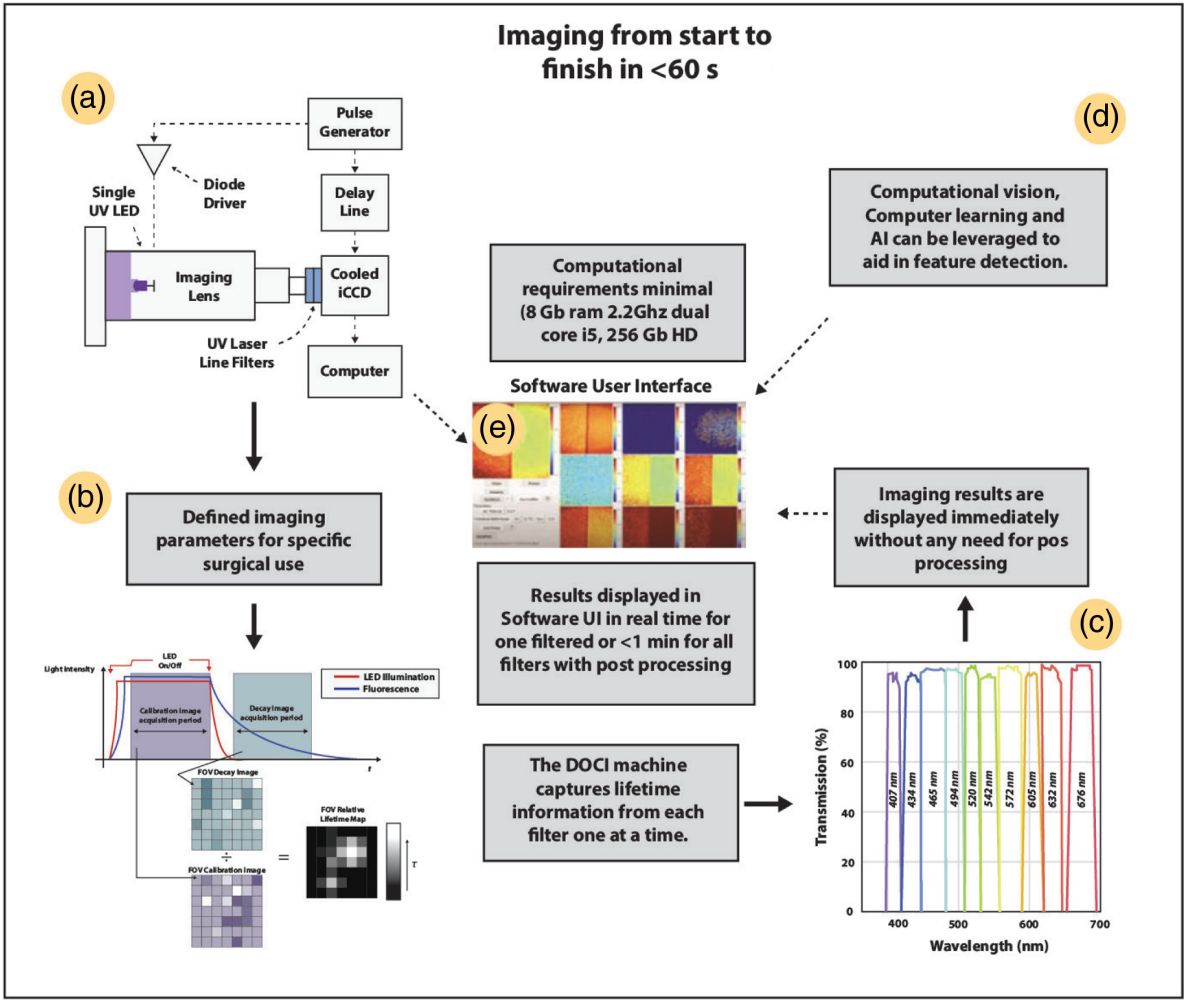
Overview of the DOCI imaging workflow. (a) Diagram illustrating the DOCI system architecture. (b) Image generation process begins with predefined imaging parameters tailored for specific surgical applications. (c) Images are acquired at the corresponding wavelengths. (d) AI-powered and computer-assisted feature detection can be integrated into the software interface. (e) User-friendly software interface allows image or video acquisition in just three clicks, with immediate display of imaging results, eliminating the need for post-processing.

The DOCI system is a widefield hyperspectral imaging platform that non-destructively captures tissue autofluorescence across 23 spectral channels (DOCI-1 … DOCI-23) using tunable excitation and synchronized emission filtering. Each acquisition produces a spatially registered 23-channel autofluorescence tensor per slide. A standardized three-click graphical user interface (GUI) automates illumination, exposure calibration, and channel sequencing, minimizing operator variability and ensuring consistent acquisition (Fig. 1). All images are automatically aligned, saved in 16-bit format to preserve true intensity contrast, and rendered immediately as multi-channel composites for real-time inspection and quality control.

### 2.2 Preprocessing and Voxel Construction

Specimens were assigned to training, validation, and test sets (n=40, 17, and 15 cases, respectively) so that no tissue of the same diagnostic class from a given individual appeared in multiple splits. Although some patients contributed more than one tissue sample, these were placed in different splits only when the underlying pathology differed. This approach prevents class-specific information from leaking across splits while still capturing natural biological variability, improving overall model robustness.

**Fig. 2:**
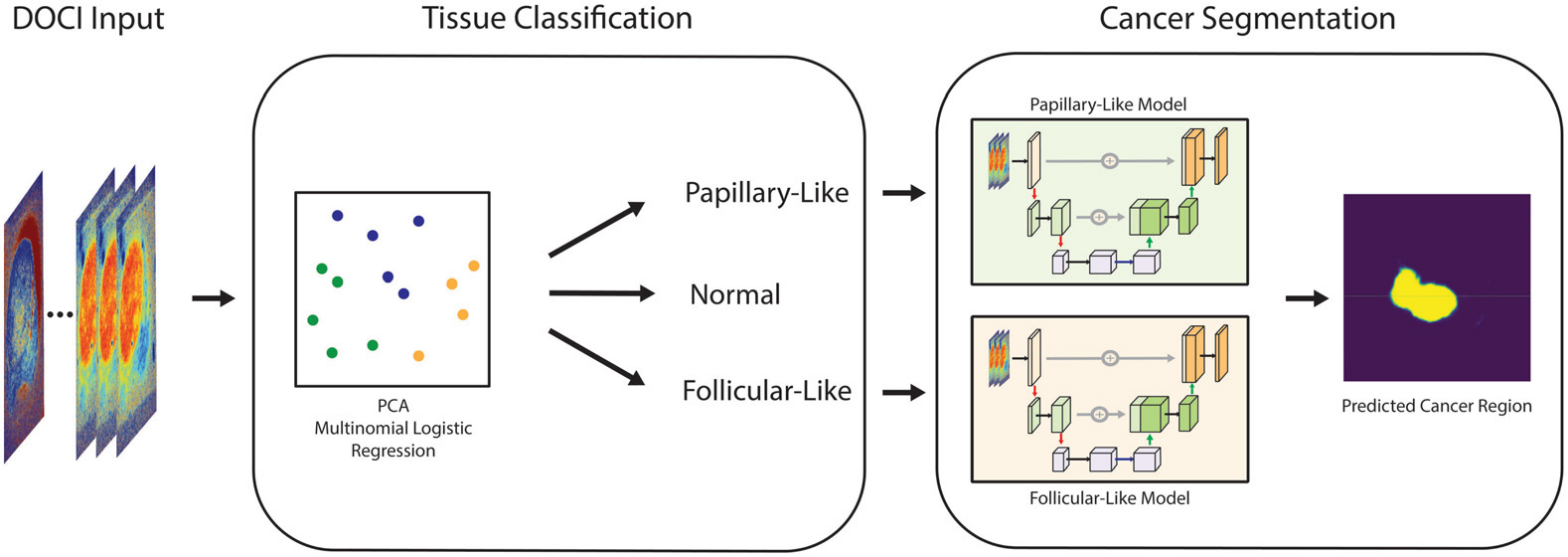
Overview of the DOCI-based tissue classification and segmentation pipeline. Each specimen is imaged with DOCI to produce a 23-channel autofluorescence voxel stack. A pixel-level PCA and logistic regression classifier generates per-pixel normal, follicular, and papillary probabilities, which are aggregated with a sliding-window majority filter to assign a specimen-level diagnostic category. This gating step determines which slides proceed to segmentation. Tumor-specific squeeze-and-excitation U-Net (SE-UNet) models then operate on voxel-only inputs to delineate cancerous regions, producing detailed probability maps and binary masks for tumor localization and quantitative analysis.

To preserve the multi-spectral structure of DOCI, each sample was represented as a voxel tensor $X\in {\mathbb{R}}^{H\times W\times 23}$ by stacking the 23 aligned channels. Each tensor was independently scaled to the range [0, 1] using min–max normalization to preserve relative spectral contrast. Masks from pathologist annotations were rasterized at the image resolution to produce binary tumor maps $Y\in {\left\{0,1\right\}}^{H\times W}$. For neural network training, tensors and masks were resized to 256×256  pixels using bilinear interpolation for image channels and nearest-neighbor interpolation for masks, preventing label contamination at edges. These transforms were applied synchronously to the image, cutoff volume, and mask to maintain precise pixel-level correspondence prior to introduction into the training workflow (Fig. 2).

### 2.3 Pixel-Level PCA Classifier and Regional Diagnostic Gating

An interpretable, slide-level diagnostic gate was established using a pixel-wise PCA and logistic regression classifier followed by regional majority voting. Pixels from tissue regions were standardized to zero mean and unit variance per channel using the training set statistics, then sampled from (1) normal tissue across all training specimens and (2) tumor ∩ tissue regions in training follicular and papillary slides, with anaplastic cases excluded. PCA was applied to the concatenated dataset, retaining eight principal components (PC) while reducing channel-level noise and autofluorescence heterogeneity. Because the DOCI data are high-dimensional relative to cohort size, dimensionality reduction was used to improve model stability and generalization in line with established approaches for small-sample, high-dimensional learning problems.^21^^,^^22^

Each PC map was transformed into a multi-scale contextual feature stack composed of (1) raw PCs; (2) Gaussian-blurred versions at σ=1, 2; and (3) gradient-magnitude maps of each blurred image to capture local texture and boundary cues. A multinomial logistic regression classifier was trained on labeled pixels to distinguish normal, follicular, and papillary tissue, excluding background pixels to prevent class dilution. The resulting probability maps P(N), P(F), and P(P) provide spatially resolved diagnostic likelihoods across tissue.

To infer specimen-level categories, a sliding-window majority filter (45- to 64-px window, 20- to 32-px stride) was applied over tissue regions. Windows with fewer than 30 tissue pixels were ignored, and the dominant tumor class was assigned if ≥85% of tissue pixels favored that class. At the specimen level, slides were labeled normal if no tumor class was detected or by the tumor type with the higher overall fraction of tumor-predicted pixels when both were present. This regional categorization step was used to gate dataset inclusion for segmentation training, and pre-filter confusion matrices were computed to evaluate the PCA-based classifier independently.

To complement point estimates of diagnostic performance, uncertainty was quantified using patient-level bootstrapped confidence intervals. For each split, patients were sampled with replacement, their slides were aggregated, and regional accuracy and class-specific recall were recomputed. Repeating this process 2000 times yielded 95% confidence intervals from the 2.5th and 97.5th percentiles of the bootstrap distribution, providing a more realistic measure of variability for this cohort size.

### 2.4 Gate-Driven Dataset Assembly and Voxel-Only Augmentation

The downstream segmentation network operated exclusively on voxel-based inputs without incorporating PCA features or probability maps. To construct the segmentation dataset, only slides categorized as either normal or the target tumor (papillary-targeted or follicular-targeted) by the gating step were retained. Optionally, anaplastic slides were also included if the classifier gate predicted the target tumor class, thereby increasing morphological diversity and improving robustness to atypical tissue features.

All data augmentations were applied synchronously to the voxel tensors, cutoff volumes, and segmentation masks to maintain pixel-wise alignment. Each augmented instance consisted of a random combination of horizontal and vertical flips, small in-plane rotations within ±15 deg around the image center, and isotropic zooming within the range [0.90, 1.10], followed by cropping or padding to maintain a uniform resolution of 256×256  pixels. Additive Gaussian noise (σ=0.001, normalized to [0, 1]) was applied to voxel inputs only, leaving masks unchanged. To mitigate class imbalance, augmentation frequency was weighted toward tumor-containing samples, with typically four augmentations per positive image and one per empty-mask image. Validation and test datasets were completely held out from augmentation to preserve the integrity of evaluation metrics.

### 2.5 Segmentation Network Architecture (SE-UNet and Voxel-Only)

**Fig. 3:**
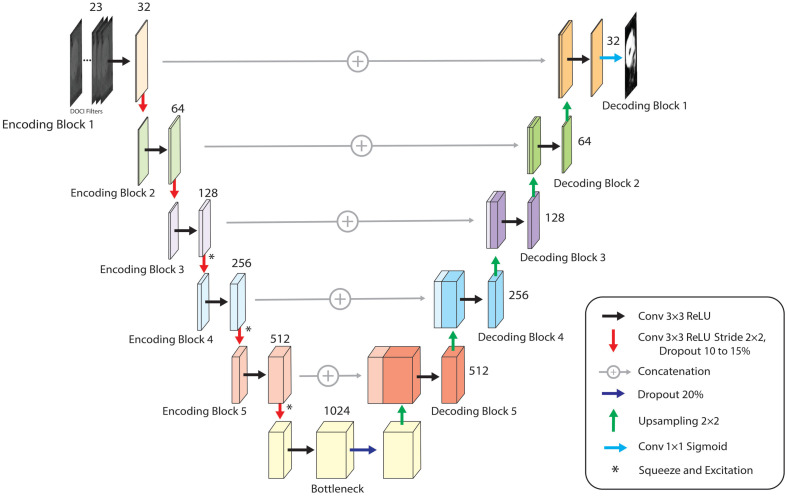
Schematic of model architecture. Multi-channel DOCI voxels pass through five encoder blocks (32 to 512 channels), each with 3×3 convolutions, batch normalization, spatial dropout, and a strided 3×3 downsampling layer. Squeeze-and-excitation modules (*) are used in encoder blocks with ≥128 channels. A 1024-channel bottleneck contains batch normalization and 20% dropout. The decoder mirrors the encoder with 2×2 upsampling, skip connections, and paired 3×3 convolutions. A final 1×1 sigmoid layer outputs the segmentation mask. Channel labels denote feature dimensions.

Segmentation was performed using a two-dimensional squeeze-and-excitation U-Net (SE-UNet) implemented in TensorFlow/Keras. The model processes a 256×256×23  voxel tensor and outputs a single-channel sigmoid probability map. The encoder consists of five convolutional stages with increasing filter depth (32 to 512), each using paired 3×3 convolutions with ReLU activation and batch normalization; squeeze-and-excitation modules are applied in deeper layers, and spatial dropout provides regularization. A 1024-filter bottleneck connects to a symmetric decoder that performs nearest-neighbor upsampling with skip connections and paired convolutional blocks. A final 1×1 convolution with sigmoid activation produces the tumor probability mask (Fig. 3). No PCA or contextual features are used during training, as these serve only for specimen gating and visualization.

Given the strong foreground-background imbalance, a hybrid loss was used:

$$L=0.5L_{\mathrm{wBCE}}+0.5L_{\text{Focal}\text{-Tversky}}.$$

Here $L_{w\mathrm{BCE}}$ is a weighted binary cross-entropy loss with positive weights inversely proportional to tumor prevalence (capped at 5.0), and

$$L_{\text{Focal}\text{-}\text{Tversky}}=(1-TI{)}^{\gamma },$$

with

$$\mathrm{TI}=\frac{\mathrm{TP}}{\mathrm{TP}+\alpha \mathrm{FN}+\beta \mathrm{FP}},\;\alpha =0.7,\;\beta =0.3,\;\gamma =0.75.$$

The network was trained with Adam for up to 100 epochs. A global threshold $t^{*}$ was selected on the validation set by sweeping t∈[0.2,0.9] and maximizing mean Dice then fixed for test inference. Performance was evaluated using three complementary metrics: Dice (non-empty), empty penalty, and balanced Dice. Dice (non-empty) quantifies segmentation accuracy on images containing a tumor by computing the Sørensen–Dice coefficient only for cases with nonzero ground-truth foreground. The empty penalty measures false-positive behavior on images without a tumor, defined as 1−FP rate, where the FP rate is the fraction of predicted-positive pixels within the evaluated region. Balanced Dice provides a single summary measure under strong class imbalance and is calculated as the mean of the Dice (non-empty) and the empty penalty. Qualitative comparisons included PCA-based tumor probability maps P(tumor), CNN predictions, ground-truth masks, and final thresholded outputs, shown in consistent test-set order for interpretability.

### 2.6 Segmentation Network Architecture (SE-UNet and Voxel-Only)

To quantify the contribution of individual DOCI channels and derive a compact spectral subset, we performed a systematic filter-ablation study on the validation set for each tumor-specific SE-UNet (papillary- and follicular-targeted). For a trained model, baseline Dice and IoU were computed by predicting tumor probability maps from the full validation voxel stack and thresholding at the global operating point $t^{*}$ defined in Sec. 2.5. We then re-evaluated performance after disrupting one DOCI channel at a time while holding all others fixed. In the primary analysis, disruption was implemented by spatially permuting pixels within that channel independently for each image, preserving its global intensity distribution but destroying spatial structure. This procedure was repeated three times per channel with different random seeds, and Dice and IoU were averaged across repeats; we additionally confirmed that simple channel zeroing produced similar importance rankings.

For each channel c, we computed the mean change in Dice and IoU relative to baseline ($\Delta {\text{Dice}}_{c}$ and $\Delta {\mathrm{IoU}}_{c}$) as a measure of importance. Channels were ranked in descending order of $\Delta {\text{Dice}}_{c}$, with $\Delta {\mathrm{IoU}}_{c}$ used to break ties. The six most impactful channels were selected separately for the papillary and follicular SE-UNets, and the union of these sets (up to 12 unique DOCI channels out of 23) defined the reduced spectral subset; all remaining channels were discarded by slicing the voxel tensors to retain only the selected indices. To test whether this subset supported accurate gating and segmentation, we retrained the entire pipeline using only the reduced channels. The PCA and contextual logistic-regression classifier (Sec. 2.3) was refit with identical pixel sampling, PCA, and feature construction procedures but applied to reduced-channel voxels. Likewise, the voxel-only SE-UNet (Sec. 2.5) was reinitialized and trained from scratch on 256×256 augmented tiles built from the reduced tensors, using the same data splits, augmentation schedule, optimizer, hybrid loss, and early-stopping settings. Performance of the reduced-channel models was then compared directly with the full-channel baseline using the same validation- and test-set metrics, enabling a fair evaluation of how much spectral redundancy could be removed without materially degrading diagnostic or segmentation accuracy.

## 3 Results

### 3.1 Regional Categorization (Gate)

**Fig. 4:**
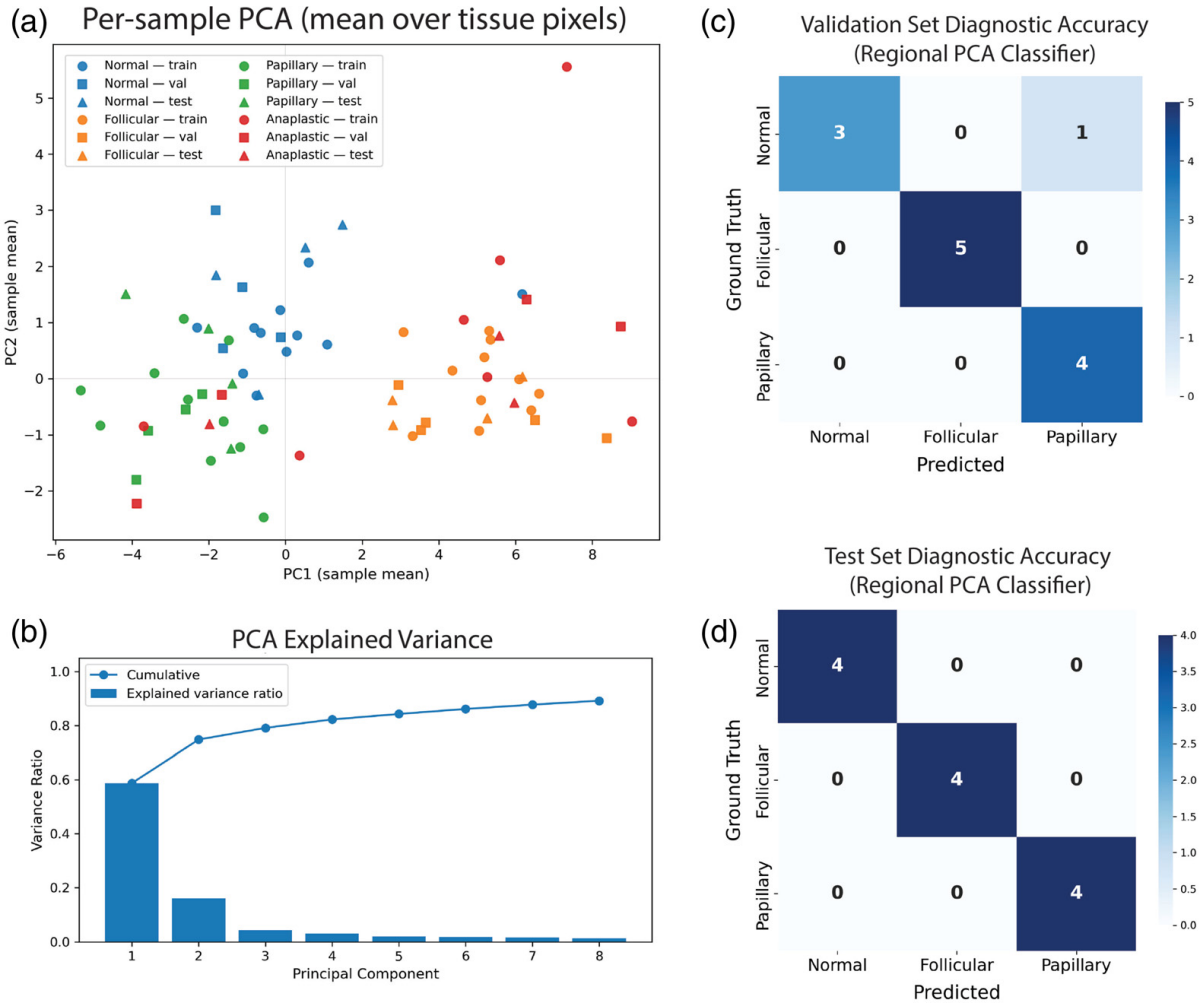
PCA characterization and diagnostic performance of the pixel-level PCA classifier. (a) Mean PC1–PC2 embeddings for each specimen, colored by diagnostic class and marked by train/val/test membership, showing clear separation of normal and papillary tissues with partial overlap between follicular and papillary cases. (b) PCA explained variance (training set only), demonstrating that the first two components capture most DOCI spectral variance. (c) Validation and (d) test set confusion matrix for regional slide-level categorization.

PCA performed on the training set revealed that the first two components captured the majority of spectral variability across DOCI channels, with PC1 explaining 59% and PC2 an additional 16% of the variance [Fig. 4(b)]. Subsequent components each contributed <5%, indicating substantial redundancy among adjacent spectral channels. When aggregated at the specimen level, the mean PC1 and PC2 embeddings of normal, follicular, and papillary tissues formed partially separable clusters with consistent morphology across training, validation, and test sets [Fig. 4(a)]. These averaged trends reflect the underlying pixel-level structure shown in Fig. S1 in the Supplementary Material, and the pixel-wise prediction patterns, illustrated in Fig. S3 in the Supplementary Material, further demonstrate that PCA features capture meaningful class-dependent contrast despite localized over- or under-prediction.

The pixel-wise PCA and logistic regression classifier produced highly discriminative specimen-level predictions after regional majority voting. Validation accuracy reached 92.3% (12/13 correct), misclassifying only one normal specimen as papillary [Fig. 4(c)], and the corresponding patient-level bootstrap estimate indicated an accuracy of 0.923 with a 95% confidence interval of 0.769 to 1.000. Performance on the held-out test set remained perfect, with 100% accuracy (12/12 correct) and a bootstrapped 95% confidence interval of 1.000 to 1.000 [Fig. 4(d)]. Training set performance was similarly strong, with a patient-level bootstrapped accuracy of 0.909 (95% CI: 0.788 to 1.000). Together, these results confirm that DOCI spectral features, even at 5-μm section thickness, contain sufficient biochemical contrast for reliable diagnostic categorization and robustly support the gating step used for downstream segmentation.

### 3.2 Segmentation Performance of Full-Channel SE-UNet Models

Voxel-only SE-UNet models trained separately for papillary and follicular carcinoma using all 23 DOCI spectral channels demonstrated strong and consistent segmentation performance across training, validation, and held-out test sets (Table 1).

**Table 1:** Segmentation performance of voxel-only SE-UNet models for papillary and follicular thyroid cancer using either all 23 DOCI channels or the reduced 12-channel subset. Metrics are reported for the training, validation, and held-out test sets. Dice (non-empty) reflects segmentation accuracy on images containing tumor, empty penalty quantifies false-positive suppression on tumor-absent slides, and balanced Dice is the mean of these two metrics, summarizing overall performance under strong class imbalance.

Model	Set	Model performance		
		Dice (non-empty)	Balanced Dice	Empty penalty
Papillary 23 channels	Train	0.826	0.910	0.995
	Val	0.807	0.899	0.990
	Test	0.829	0.914	0.999
Follicular 23 channels	Train	0.708	0.852	0.996
	Val	0.652	0.825	0.999
	Test	0.618	0.809	1.000
Papillary 12 channels	Train	0.919	0.960	1.000
	Val	0.750	0.859	0.969
	Test	0.672	0.831	0.990
Follicular 12 channels	Train	0.750	0.827	0.904
	Val	0.713	0.784	0.854
	Test	0.762	0.815	0.867

The full-channel papillary model produced the strongest overall performance across all evaluated metrics. On the validation set, the model achieved a Dice (non-empty) of 0.807, indicating reliable pixel-level detection of papillary areas. Held-out test performance remained similarly high, with a Dice (non-empty) of 0.829, a balanced Dice of 0.914, and near-perfect empty-image penalties (0.999), demonstrating excellent specificity and minimal false-positive behavior.

The per-tile Dice distribution showed a median of 0.832, reflecting minimal outlier degradation and stable behavior across heterogeneous papillary cases.

Segmentation of follicular areas, known to exhibit weaker intrinsic autofluorescence contrast and more heterogeneous morphology, was more challenging. On the validation set, the model achieved a Dice (non-empty) of 0.652, with test performance of 0.618. Nevertheless, the model produced a high balanced Dice (0.809), driven in part by extremely strong empty-image penalties (∼1.000), reflecting that the model rarely produced spurious detections.

**Fig. 5:**
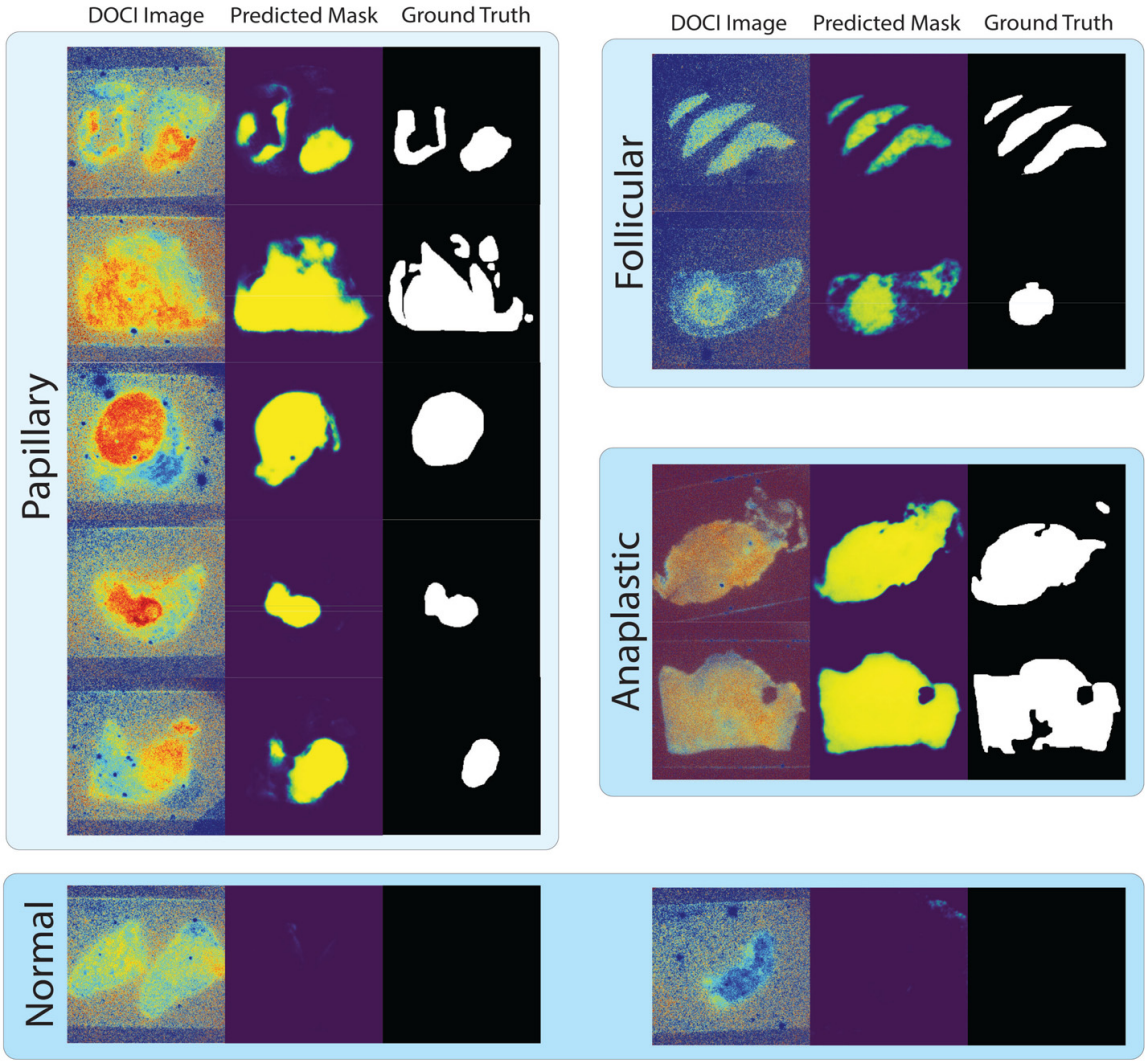
Qualitative segmentation results from the full 23-channel voxel-only SE-UNet models. Each panel shows the DOCI input image (left), predicted tumor probability mask (middle), and corresponding ground-truth annotation (right). Papillary examples and normal tissue examples are segmented using the papillary-targeted model, whereas follicular and anaplastic examples are segmented using the follicular-targeted model (anaplastic images were excluded from PCA training but included here to assess cross-class generalization).

The prediction results are shown in Fig. 5, which illustrate the model’s ability to accurately localize tumor regions across papillary, follicular, anaplastic, and normal specimens. Together, these results demonstrate that full-spectral DOCI imaging contains sufficient information for robust tumor-type specific segmentation, particularly for papillary carcinoma.

### 3.3 Impact of Reduced-Channel DOCI Inputs After PCA-Guided Spectral Ablation

To determine whether all 23 spectral channels were necessary for accurate segmentation, we conducted a systematic channel-importance analysis. For each SE-UNet, channels were perturbed individually (via spatial permutation) to quantify performance degradation (ΔDice and ΔIoU), revealing distinct channel-sensitivity patterns for papillary and follicular models (Fig. S4 in the Supplementary Material). The top six most influential channels for each tumor type were then combined to produce a 12-channel reduced spectral subset. Both the PCA gate and SE-UNet models were retrained from scratch using only this reduced input. Performance of the papillary model showed moderate degradation compared with the full 23-channel baseline (Dice = 0.672 versus 0.829, balanced Dice = 0.831 versus 0.914) (Table 1), suggesting that papillary tissue relies on a broader span of the DOCI spectrum than follicular tissue, consistent with their more distinct intrinsic autofluorescence signatures observed during acquisition.

Surprisingly, the follicular model exhibited improved Dice performance with reduced-channel inputs compared with the full model. Test Dice increased from 0.618 to 0.762, suggesting that reducing spectral redundancy improved generalization and reduced overfitting to low-utility channels. Balanced Dice remained effectively unchanged (0.809 versus 0.815), and boundary performance increased modestly (Table 1). These findings imply that follicular carcinoma segmentation is dominated by a small set of high-value spectral features and may benefit from reduced spectral dimensionality.

Overall, the reduced 12-channel subset preserved most segmentation performance across both tumor types, despite cutting input dimensionality nearly in half. The contrasting responses, improved performance for follicular versus moderate declines for papillary, highlight that (1) papillary segmentation benefits from more complete spectral coverage, reflecting its richer biochemical contrast profile and (2) follicular segmentation depends on a narrow set of discriminative spectral bands. The reduced-channel pipeline nonetheless maintained high diagnostic utility, demonstrating that substantial spectral redundancy exists in the DOCI acquisition, and that compact spectral subsets may enable faster, lower-cost implementations.

## 4 Discussion

Accurate intraoperative assessment of thyroid cancer remains a central challenge in endocrine oncology, where surgeons must balance complete tumor resection against the preservation of critical neck structures. Standard histopathology provides high-quality diagnostic and margin information but only after excision and substantial processing time. DOCI presents a fundamentally different paradigm: an immediate, label-free, widefield characterization of intrinsic tissue autofluorescence that can be acquired directly from fresh surgical specimens. In this work, we demonstrate that DOCI, when paired with a principled machine learning pipeline, provides reliable diagnostic categorization and high-resolution tumor segmentation across thyroid cancer subtypes.

Our PCA-based diagnostic gate achieved strong slide-level accuracy on both validation and test sets, illustrating that the spectral structure of DOCI images encodes sufficient biochemical contrast to separate normal, follicular, and papillary tissues. The PCA and logistic regression framework was intentionally designed to be interpretable, computationally lightweight, and compatible with real-time deployment. The clear separation observed in PC space, along with the near-perfect confusion matrices, suggests that DOCI’s spectral signatures offer a stable foundation for downstream tasks even at 5  μm section thickness.

For tumor localization, voxel-only SE-UNet models trained on the full 23-channel DOCI stacks achieved consistently high segmentation accuracy, particularly for papillary carcinoma. High Dice (non-empty), near-perfect empty-penalty scores, and strong balanced Dice values confirm that the models not only detect tumor regions reliably but also avoid spurious predictions on tumor-absent slides, a key requirement for intraoperative applications. Qualitative visualizations further highlight the models’ ability to delineate heterogeneous tumor morphologies, including cases with complex boundaries or mixed tissue composition.

Our spectral ablation study provides new insight into the relationship between DOCI’s multispectral information and segmentation performance. Although papillary regions benefited from the full spectral range, follicular regions demonstrated improved generalization when trained on a compact 12-channel subset derived from PCA-guided importance ranking. This asymmetry suggests that papillary carcinoma expresses richer or more spatially diffuse autofluorescence signatures, whereas follicular carcinoma may rely on a smaller number of high-value spectral bands. Importantly, the reduced-channel pipeline preserved much of the diagnostic performance while cutting input dimensionality nearly in half, highlighting that substantial redundancy exists in the full DOCI acquisition. These findings have practical implications for future system design: optimized DOCI hardware using only a strategically selected subset of wavelengths may reduce acquisition time, cost, and system complexity without compromising diagnostic utility.

Clinically, this work illustrates how DOCI combined with machine learning could augment current diagnostic pathways. A rapid, label-free approach to distinguishing thyroid cancer subtypes and localizing tumor margins could support several use cases: improving the interpretive confidence of FNA biopsies, guiding intraoperative decision-making, reducing unnecessary resections, and enabling margin assessment directly in the operating room. The ability to maintain high specificity in normal tissue, even with reduced spectral dimensionality, strengthens DOCI’s potential role as a real-time adjunct to surgical pathology.

Future work should validate these models across larger, multi-institutional datasets and further examine cross-class generalization, particularly for rare but clinically urgent subtypes such as anaplastic carcinoma. In addition, expanding DOCI to *in vivo* or near-real-time imaging scenarios, coupled with on-device machine learning, may pave the way for next-generation optical guidance tools in head and neck surgery. Overall, this study provides strong evidence that DOCI’s rich spectral information, when harnessed through carefully designed computational models, can substantially enhance the accuracy, speed, and clinical utility of thyroid cancer diagnostics.

## 5 Conclusion

This study demonstrates that machine learning enhanced DOCI offers a compelling framework for thyroid cancer diagnosis and intraoperative guidance. By combining DOCI’s rich, label-free spectral information with an interpretable PCA and logistic regression gate and tumor-specific SE-UNet segmentation models, we achieved accurate slide-level categorization and robust pixel-level delineation of papillary and follicular thyroid carcinoma.

Our findings show that full 23-channel DOCI acquisitions provide particularly strong performance for papillary lesions, whereas a PCA-guided 12-channel subset can maintain, or even improve, follicular segmentation accuracy, revealing distinct spectral dependencies between tumor subtypes and highlighting substantial redundancy in the full spectral stack. This balance between spectral richness and dimensionality reduction has practical implications for future DOCI hardware and acquisition protocols, potentially enabling faster, lower-cost systems without sacrificing diagnostic utility.

Taken together, these results lay a strong foundation for integrating DOCI with existing workflows such as fine-needle aspiration assessment and intraoperative margin evaluation. With further validation in larger, multi-institutional cohorts and translation to real-time clinical implementations, DOCI coupled with targeted machine learning models has the potential to become a powerful tool for precision diagnosis, surgical decision support, and improved outcomes in head and neck oncology.

## Supplementary Material

10.1117/1.BIOS.3.1.015001.s01

## Data Availability

The data that support the findings of this study are openly available in GitHub at https://github.com/tylervasse/DOCI-Prediction.
