# Supplementary material for: Machine-learning-based tumor segmentation and classification using dynamic optical contrast imaging for thyroid cancer
Source: Biophotonics Discov. 2026 Jan 2;3(1):015001. doi: 10.1117/1.BIOS.3.1.015001 (PMC13052498; doi:10.1117/1.BIOS.3.1.015001)
Supplement: Supplementary file 1 [file BIOS_003_015001_SD001.pdf]

Supplementary Figures:

SUPPLEMENTARY TABLE 1. DOCI FILTERS	
**Filter**	**Wavelength**
**Filter 1**	400nm/Long Pass
**Filter 2**	413nm/10nm
**Filter 3**	420nm/10nm
**Filter 4**	430nm/10nm
**Filter 5**	440nm/10nm
**Filter 6**	450nm/10nm
**Filter 7**	460nm/10nm
**Filter 8**	467nm/10nm
**Filter 9**	470nm/10nm
**Filter 10**	473nm/10nm
**Filter 11**	480nm/10nm
**Filter 12**	486nm/10nm
**Filter 13**	488nm/10nm
**Filter 14**	492nm/10nm
**Filter 15**	500nm/10nm
**Filter 16**	510nm/10nm
**Filter 17**	520nm/10nm
**Filter 18**	532nm/10nm
**Filter 19**	546nm/10nm
**Filter 20**	560nm/10nm
**Filter 21**	580nm/10nm
**Filter 22**	589nm/10nm
**Filter 23**	594nm/10nm

Supplementary Table 1. *List of DOCI filters and their corresponding wavelength specifications.* Each filter captures autofluorescence signals within a specific spectral band, enabling the generation of multispectral DOCI images for downstream classification and segmentation. Filters span the visible spectrum from 400 nm (long-pass) to 594 nm with 10 nm bandwidths.

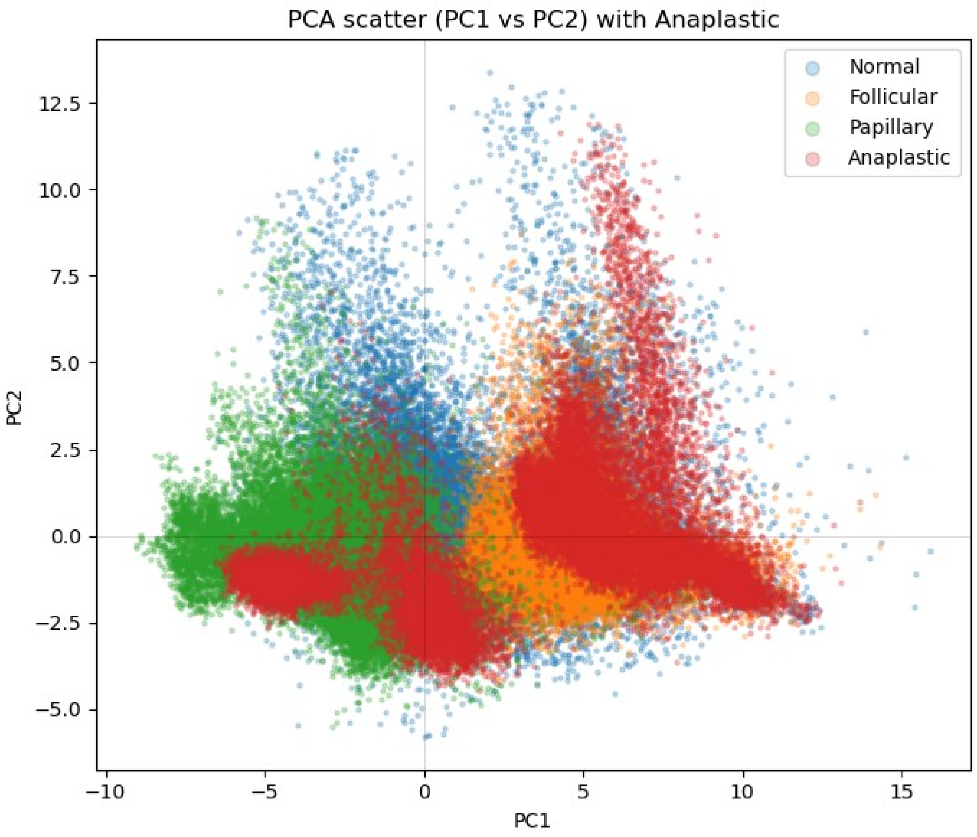

Supplementary Fig.1. *PCA plot of pixel-level DOCI data, visualizing the distribution of tissue types in a two-dimensional space.* Each point represents a pixel, color-coded by tissue type: Normal (blue), Follicular (yellow), Papillary (green), and Anaplastic (red). The clustering patterns highlight distinct groupings for Normal, Follicular, and Papillary tissues, while Anaplastic samples exhibit overlapping distributions, reflecting their heterogeneous nature.

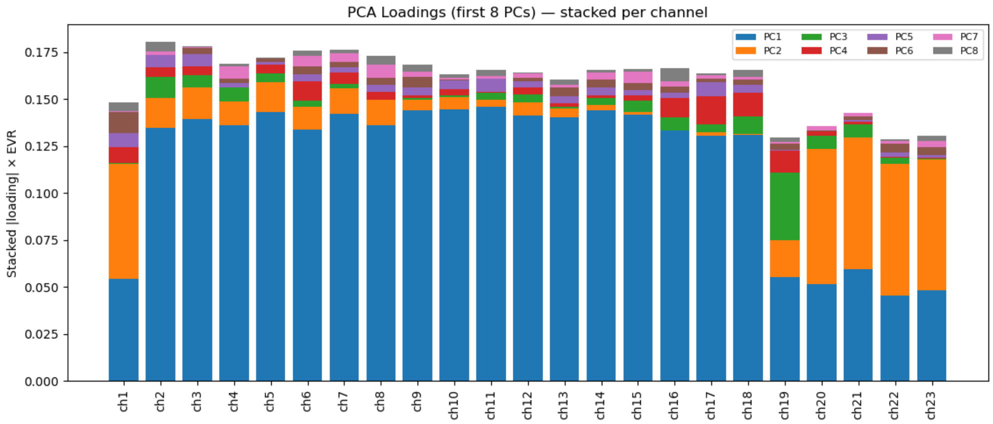

Supplementary Fig. 2. *Stacked absolute PCA loadings for the first eight principal components (PC1–PC8) across all 23 DOCI channels.* For each channel, bar height reflects the cumulative contribution of that channel to the variance captured by the first eight PCs, with individual colors indicating each component's loading weighted by its explained variance ratio. Channels 1–18 exhibit relatively uniform cumulative contributions, whereas channels 19–23 show more variable distributions across components, including elevated contributions from PC2 and PC3.

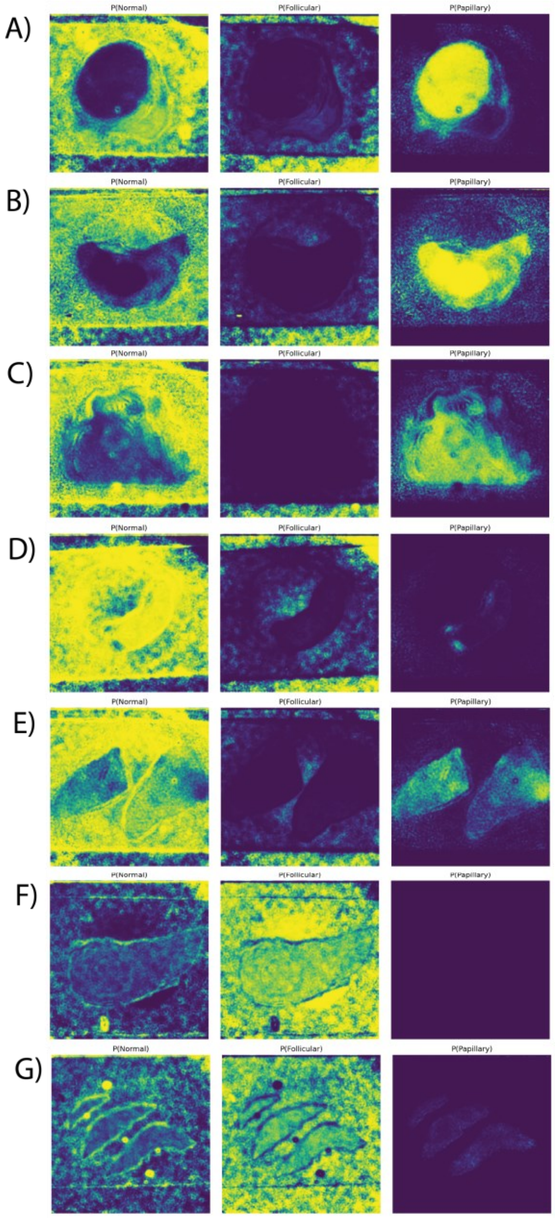

Supplementary Fig. 3. *Pixel-level PCA classifier outputs across representative Normal, Follicular, and Papillary specimens.* Rows A–C show true Papillary cases, where the PCA-based pixel predictions correctly highlight Papillary regions, although mild over-prediction is visible in panel B. Panels D and E depict Normal samples; while overall predictions are accurate, some background regions show elevated response. Panels F and G illustrate Follicular cases, where the PCA classifier exhibits clear confusion between background and tumor regions, including notable over-prediction. These limitations highlight that while PCA-based contextual features provide coarse diagnostic cues, they are insufficient for reliable pixel-level delineation, motivating the need for more expressive, spatially aware segmentation models such as the SE-UNet used in the main analysis.

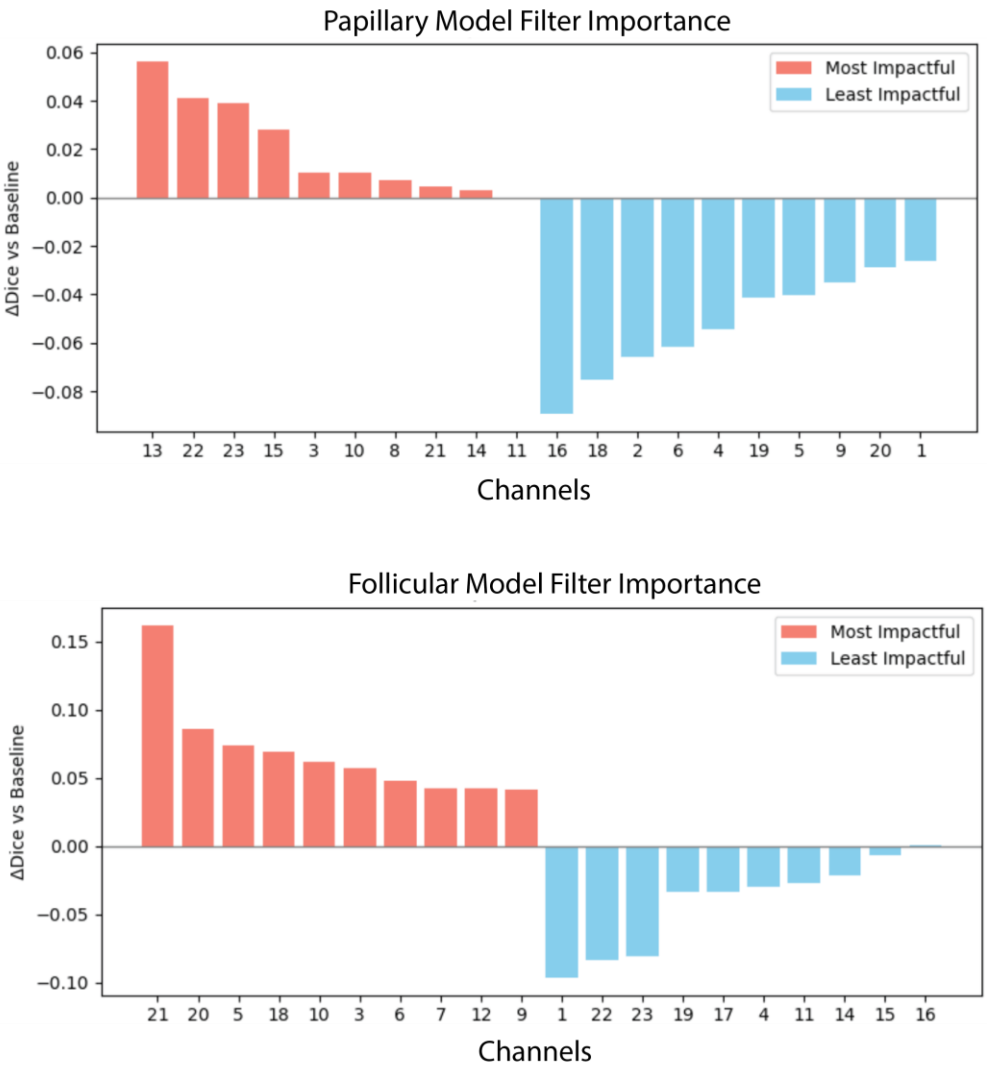

Supplemental Fig. 4. DOCI channel-ablation analysis for Papillary (top) and Follicular (bottom) SE-UNet models. Each bar shows the change in segmentation Dice score (ΔDice) when a single DOCI channel is spatially permuted while all others remain intact. Positive ΔDice values (red) indicate channels whose disruption most reduces performance, identifying them as high-impact spectral features. Negative ΔDice values (blue) correspond to channels with minimal influence on the model. Papillary segmentation depends on a broader range of informative channels, whereas Follicular segmentation is dominated by a smaller set of highly discriminative wavelengths. These distinct spectral patterns motivated the construction of a reduced-channel subset used in the main analysis.
